# A Two-Way Mendelian Randomization Analysis on the Link between Thyroid Activity Function and Coronary Atherosclerosis

**DOI:** 10.31083/j.rcm2512453

**Published:** 2024-12-24

**Authors:** Le-tai Li, Jia-jie Leng, Yu-xiang Luo, Rong-jia Liu, Zhuo-xuan Song, Meng Ye, Zhen-han Li, Zhen-rui Cao, Ying-jiu Jiang, Hong-tao Tie

**Affiliations:** ^1^Department of Cardiothoracic Surgery, The First Affiliated Hospital of Chongqing Medical University, 400016 Chongqing, China; ^2^School of Traditional Chinese Medicine, Chongqing Medical University, 400016 Chongqing, China; ^3^Department of Endocrinology, Chongqing Traditional Chinese Medicine Hospital, 400021 Chongqing, China

**Keywords:** Mendelian randomization, coronary atherosclerosis, thyroid function, endocrine dysfunction, genome-wide association studies

## Abstract

**Introduction:**

Coronary atherosclerosis serves as the primary pathological etiology underlying coronary artery disease (CAD). Thyroid hormones show potential as risk factors, aside from the main standard modifiable cardiovascular risk factors (SMuRFs). This research seeks to elucidate the link between thyroid activity and coronary atherosclerosis.

**Methods:**

Single nucleotide polymorphisms (SNPs) linked to hypothyroidism (N = 213,990), Graves’ disease (GD) (N = 190,034), other hyperthyroidism types (N = 190,799), thyroid-stimulating hormone (TSH) (N = 271,040), free thyroxine (FT4) (N = 119,120), and coronary atherosclerosis (N = 360,950) were retrieved from the IEU OpenGWAS, Finngen R9, and ThyroidOmics Consortium databases. Following the application of strict criteria to eliminate linkage disequilibrium, palindromic sequences, and heterozygous alleles, a bidirectional Mendelian Randomization (MR) analysis was conducted between the thyroid gland and coronary atherosclerosis using inverse variance weighting (IVW), weighted median (WM), and MR-Egger techniques. For sensitivity analysis, Cochran’s Q test, leave-one-out method, and MR-Egger regression analysis were employed.

**Results:**

The forward MR analysis indicates that genetic predispositions such as hypothyroidism (OR = 1.07; 95% CI 1.01–1.12; IVW-*p* = 0.021), Graves’ disease (OR = 1.04; 95% CI 1.01–1.07; IVW-*p* = 0.002), and other forms of hyperthyroidism (OR = 1.05; 95% CI 1.01–1.10; IVW-*p* = 0.021) elevate the likelihood of developing coronary atherosclerosis. Additionally, no discernible evidence of a causality between FT4 or TSH, and coronary atherosclerosis (IVW-*p* > 0.05) was found. Coronary atherosclerosis is not related to increased risk of five thyroid function phenotypes in reverse MR analysis. The sensitivity analysis provided relatively reliable evidence to reinforce the validity of our findings.

**Conclusions:**

Our findings are an investigation of the causality between thyroid function and coronary atherosclerosis. This study pinpointed potential heart disease risks linked to coronary atherosclerosis and offered additional understanding for defining SMuRFs in CAD.

## 1. Introduction

Cardiovascular disorders (CVD) have become the global major cause of death [1]. There has been a significant increase in coronary artery disease (CAD), a significant constituent of CVD in recent years [2]. Globally, the annual all-cause mortality due to CAD has reached 7.2 million, and approximately 126 million individuals are affected [3]. Over time, CAD can develop into stable angina, acute coronary syndrome (ACS), heart failure (HF), and even sudden cardiac death (SCD) [4]. Coronary atherosclerosis, characterized by the formation of plaque and vascular stenosis, diagnosed by angiography, is the primary pathophysiologic mechanism leading to coronary ischemia and the development of CAD [5]. The rupture of an unstable plaque can lead to intravascular thrombosis, resulting in ACS [6]. Thus, coronary atherosclerosis risk factors for CAD need to be investigated. The primary major standard modifiable cardiovascular risk factors (SMuRFs) include high blood pressure, abnormal lipid levels, diabetes, and smoking. Yet, a growing number of individuals are developing CAD without traditional risk factors. Notably, there wasa substantial rise from 11% in 2006 to 27% in 2014 among ST elevation myocardial infarction individuals treated at an Australian institution [7]. A recent comprehensive review in the Journal of the American College of Cardiology outlines the SMuRFless CAD clinical pathway, aimed at identifying various risk factors beyond SMuRFs [8]. This research aimed to complete and address undefined risk factors in response to the implementation of SMuRFless in CAD.

Thyroid function disorders encompass two major forms, hyperthyroidism and hypothyroidism. Both conditions are linked to heart attacks, strokes, and heart failure [9, 10, 11]. Research indicates that individuals with hypothyroidism exhibit a greater occurrence of various higher prevalence of several SMuRFs, such as high cholesterol and diabetes [12], while those with hyperthyroidism experience a higher rate of hypertension and increased platelet clumping [13, 14]. As a result, thyroid activity could be a contributing factor to CAD. Currently, the relationship of thyroid dysfunction to CAD is based primarily on observational studies, which are subject to confounding factors and ambiguity of causality.

Mendelian randomization (MR) analysis is a robust technique that employs genetic variants as instrumental variables (IVs) to explore possible causal links between clinical exposure and disease outcomes. The presence of unobserved confounders and the potential for reverse causality can be addressed by implementing a conceptual random allocation of alleles [15]. At conception, genetic alleles are distributed randomly, allowing for the simulation of randomized controlled trials to determine causal links [16]. Numerous single nucleotide polymorphisms (SNPs) linked to thyroid function traits and CAD have been discovered through genome-wide association studies (GWAS) [17, 18]. The use of MR analysis makes it possible to investigate genetic and potentially causal links between thyroid function and coronary atherosclerosis. As far as we know, no MR research has explored the causal relationship between various thyroid conditions (such as hyperthyroidism, hypothyroidism, thyroid hormones, and thyroid-stimulating hormone) and CAD. This research seeks to determine a cause-and-effect link between thyroid issues and to offer further support for the adoption of SMuRFless in CAD.

## 2. Method

### 2.1 Study Design and Data Sources

**Fig. 1.**
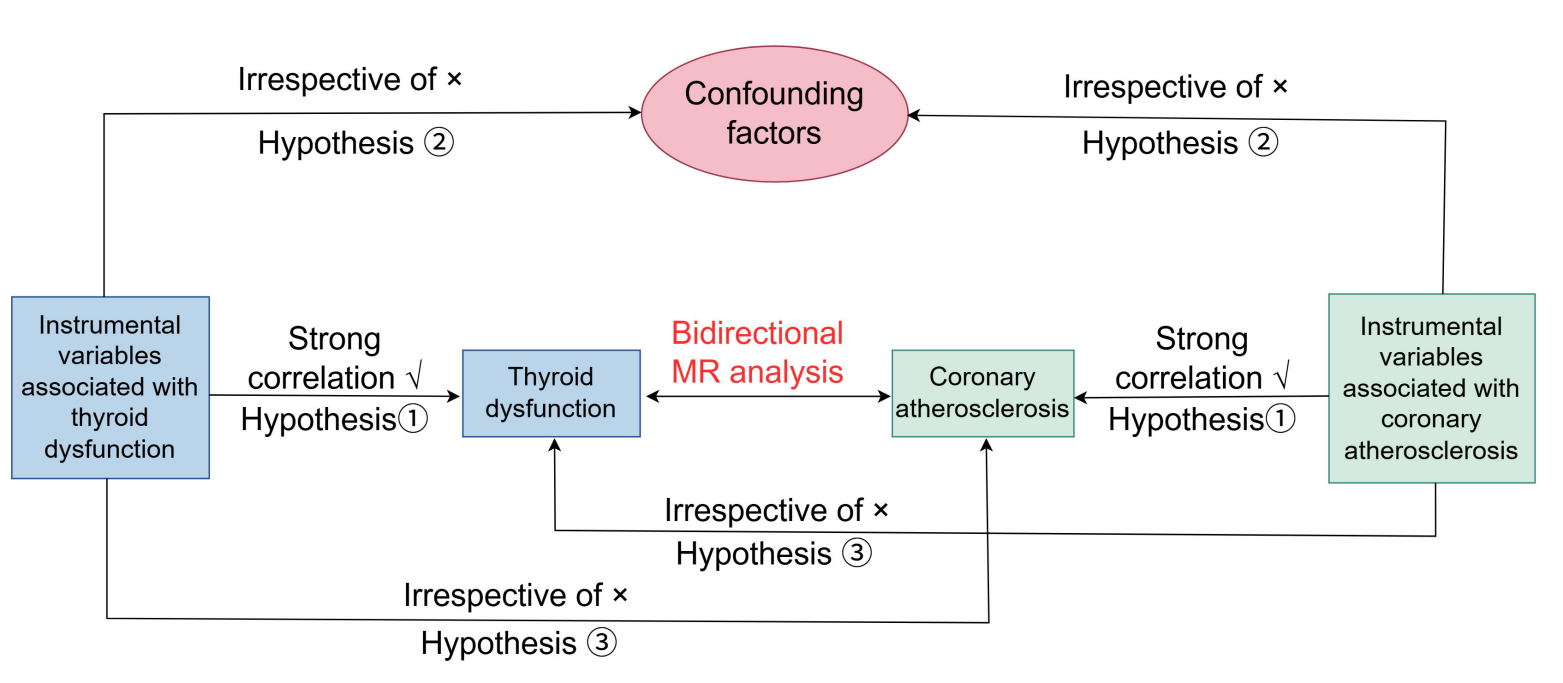
**The subsequent diagram depicts the comprehensive design and flowchart of the current research study**. MR, Mendelian Randomization.

The flowchart of this bidirectional MR study design is presented in Fig. 1. The study examines the relationship between thyroid function and coronary atherosclerosis by utilising summary data from various GWAS. To minimize potential confounding factors due to population stratification, this MR analysis exclusively involved participants of European descent. Information on coronary atherosclerosis was obtained from the FinnGen R9 database (https://r9.risteys.finngen.fi/endpoints/I9_CORATHER), including 360,950 participants (47,550 cases and 313,400 controls) [19]. Data on GWAS for hypothyroidism, Graves’ disease (GD), and various hyperthyroidism forms were sourced from the IEU OpenGWAS database (https://gwas.mrcieu.ac.uk), including 26,036, 2350, and 3115 cases, respectively, along with 187,684 controls [20]. In the end, the total sample size was 213,990 for hypothyroidism, 190,034 for Graves’ disease, and 190,799 for hyperthyroidism. Furthermore, we compiled information on thyroid-stimulating hormone (TSH) and free thyroxine (FT4) from the ThyroidOmics Consortium database (https://transfer.sysepi.medizin.uni-greifswald.de/thyroidomics/), which has been featured in Nature Communications, with sample sizes of 271,040 and 119,120 respectively [21]. More detailed information on these phenotypes can be found in the **Supplementary Table 1**.

### 2.2 Independent Variable (IV) Selection

The MR framework needs to meet three fundamental criteria: (1) Genetic instruments must have a strong link to the exposure being studied (relevance criterion); (2) Instruments should not be related to any confounders affecting the exposure-outcome relationship (independence criterion); (3) Instruments should impact the outcome solely via the exposure (exclusion restriction criterion). To establish the screening threshold for SNPs associated with the exposure, rigorous standards (*p* < 5 × 10^-8^) and independence parameters (r^2^ < 0.001, kb = 10,000) were applied. By removing all SNPs with palindromic sequences to maintain consistent effect alleles, the IVs were found to be strong, indicated by an F-statistic greater than 10. F-statistic is calculated according to the formula ((N-2) × R^2^/(1-R^2^)). R^2^, derived from the equation ((2 × beta^2^ × effect allele frequency (EAF) × (1-EAF)), indicates the fraction of exposure variance explained by the genetic instrumental variables [22]. Ultimately, SNPs that were palindromic, heterozygous, or duplicated were removed, while those with strong IVs were retained.

### 2.3 Statistical Analysis

This research utilized the TwoSampleMR package within R software version 4.3.0 (R Foundation for Statistical Computing, Vienna, Austria) for statistical analysis. To examine the causal effects, methods such as inverse variance weighting (IVW), weighted median (WM), and MR Egger were employed. IVW stands as the strongest method given the strict condition that all instrumental variables are legitimate [23]. Cochran’s Q value >0.05 indicated no heterogeneity among SNPs [24]. The MR-Egger intercept test was employed to assess horizontal pleiotropy, and a *p*-value exceeding 0.05 indicated its absence [25].

If the heterogeneity of the IVs was not qualified, an IVW with random effects model was the primary effect estimate, while the fixed effects model was used if heterogeneity was qualified [26]. If the IVW method yielded significant results and both the WM and MR-Egger methods produced consistent findings in the same direction as those of IVW, a causal relationship was inferred [27]. In addition, the influence of single SNP on the overall causal effect was examined through leave-one-out analysis. In the reverse analysis, the causal relationship was analyzed using the same methods as described above.

## 3. Results

### 3.1 IV Selection

Regarding thyroid activity and its connection to coronary atherosclerosis, 53, 14, 12, 167, and 63 IVs not influenced by linkage disequilibrium (LD) were evaluated in relation to hypothyroidism, GD, various hyperthyroid conditions, TSH, FT4. In the reverse analysis, a total of 31 SNPs associated with coronary atherosclerosis were selected as IVs. F-statistics for each IV were greater than 10, suggesting no weak IV bias. **Supplementary Tables 2–7** offers a detailed catalog of SNPs linked to exposure.

### 3.2 MR and Sensitivity Analysis for Causality of Thyroid Function on Coronary Atherosclerosis

**Fig. 2.**
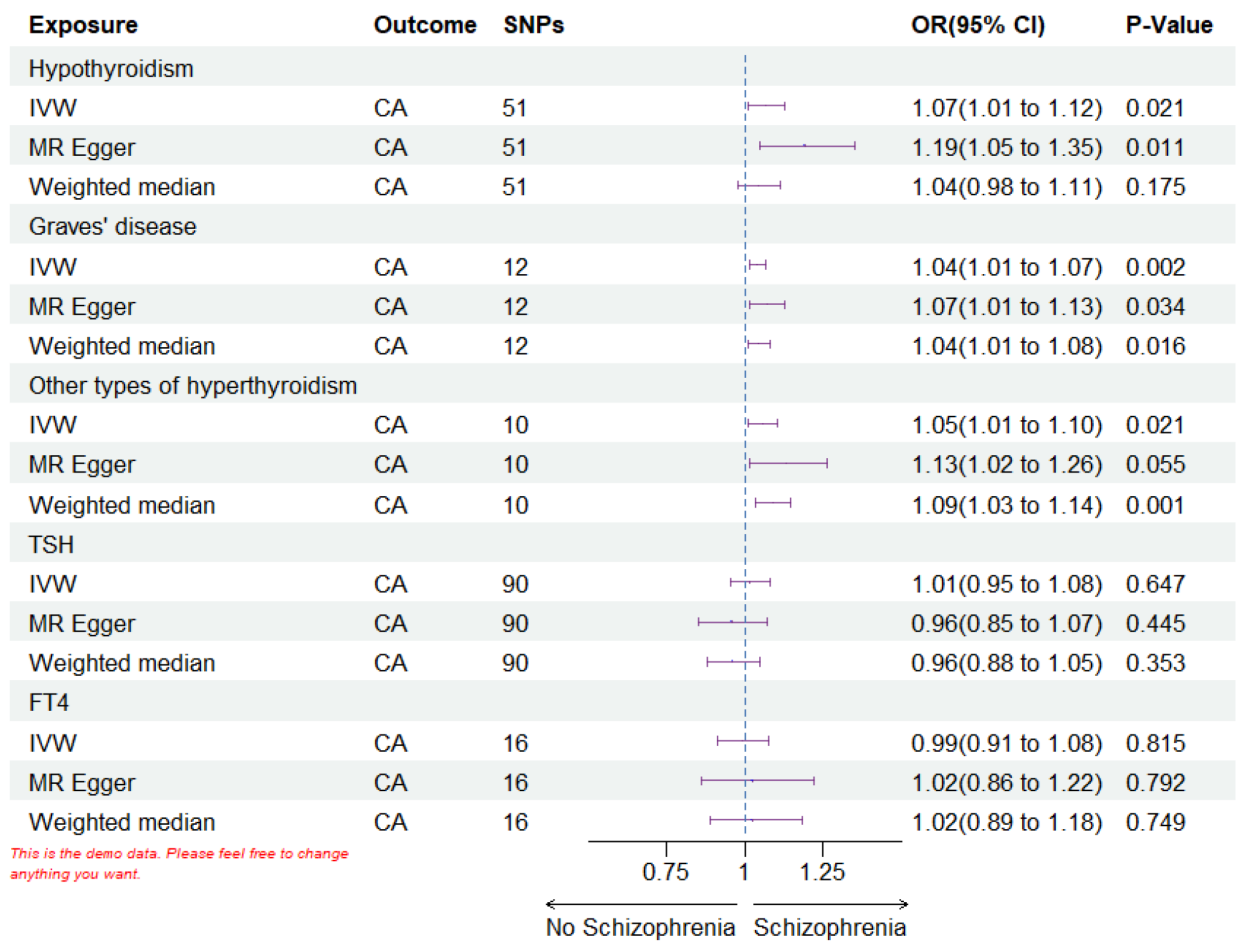
**The forest plots of forward MR analysis**. IVW, inverse variance weighted; SNPs, single nucleotide polymorphisms; OR, odds ratio; CI, confidence interval; CA, coronary atherosclerosis; FT4, free thyroxine; TSH, thyroid-stimulating hormone; MR, Mendelian Randomization. Every statistical test was conducted using a two-tailed approach. A *p*-value less than *p* < 0.05 was deemed statistically significant.

**Fig. 3.**
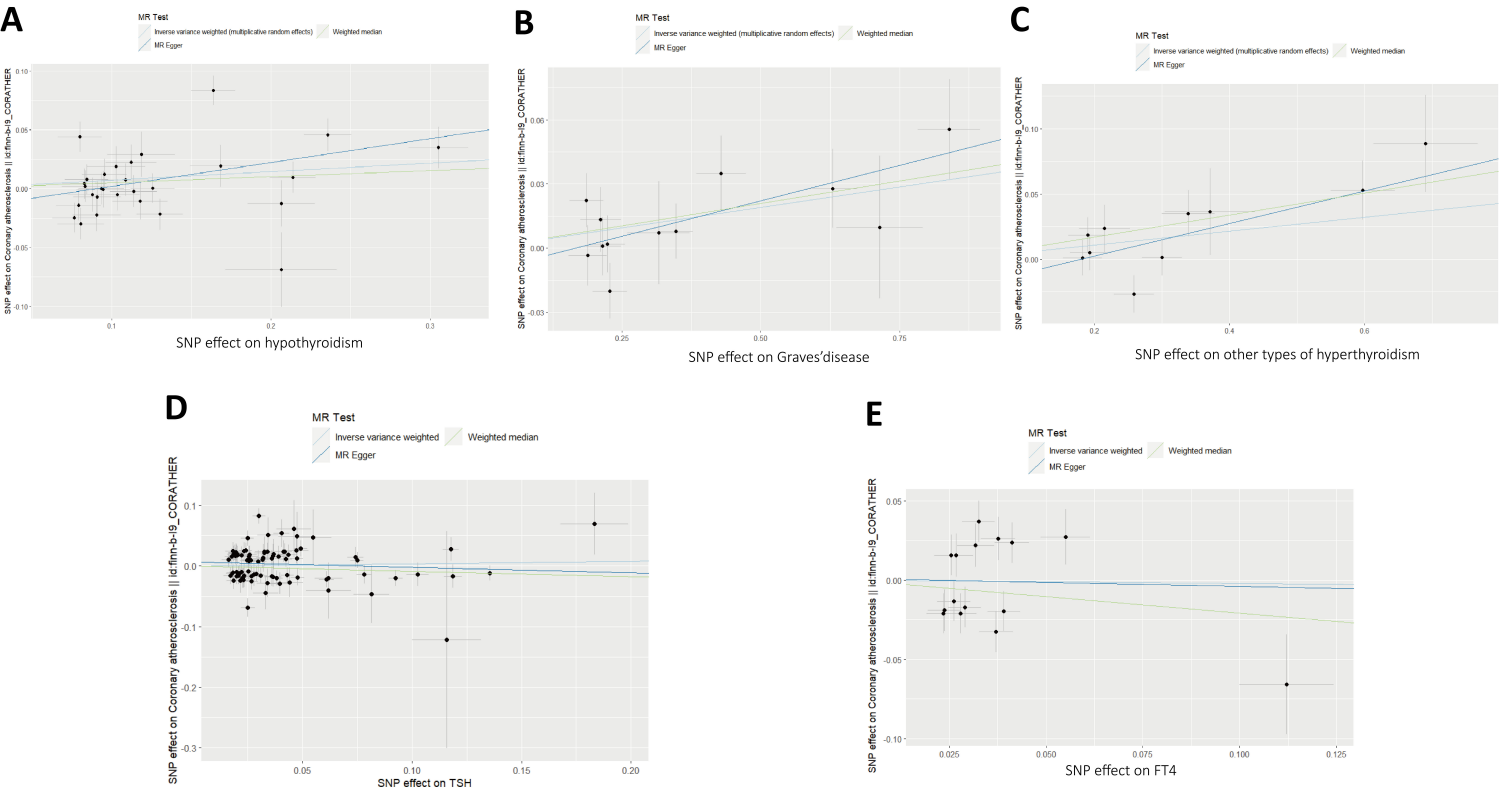
**Displays scatter plots illustrating the causal impact derived from MR analysis**. (A) The impact of hypothyroidism on coronary atherosclerosis. (B) Graves’ disease on coronary atherosclerosis. (C) The impact of other types of hyperthyroidism on coronary atherosclerosis. (D) The impact of thyroid-stimulating hormone on coronary atherosclerosis. (E) FT4 on coronary atherosclerosis. SNP, single nucleotide polymorphism; FT4, free thyroxine; TSH, thyroid-stimulating hormone; MR, Mendelian Randomization.

Among all eligible SNPs, the palindromic, heterozygous, duplicated, and weak SNPs (2, 2, 2, 77, and 47) for the assessment of five types of thyroid function were excluded. Consequently, our final set for MR analysis comprised 51, 12, 10, 90, and 16 SNPs. IVW analysis indicated a positive correlation between the risk of coronary atherosclerosis and hypothyroidism (OR = 1.07; 95% CI 1.01–1.12; *p* = 0.021), GD (OR = 1.04; 95% CI 1.01–1.07; *p* = 0.002), as well as other forms of hyperthyroidism (OR = 1.05; 95% CI 1.01–1.10; *p* = 0.021). The predictive results by MR Egger and Weight Median (OR >1) associated with the IVW analysis is shown in Fig. 2 and Table 1. In this research, the impact of TSH and FT4 on the likelihood of developing coronary atherosclerosis was found to be insignificant. No horizontal pleiotropy was observed in the scatter plots in the forward analysis (Fig. 3). However, the heterogeneity for SNPs of hypothyroidism and TSH was detected (*p* < 0.05) using the IVW random-effects model (Table 2). Conversely, for the others, the IVW fixed effects model was used.

**Table 1.** **The causality between thyroid function on coronary atherosclerosis**.

Exposure		Outcome	SNPs	OR	*p*-Value	95% CI Low	95% CI High
Hypothyroidism							
	IVW	coronary atherosclerosis	51	1.066	0.021	1.010	1.125
	MR Egger	coronary atherosclerosis	51	1.190	0.011	1.048	1.352
	Weighted median	coronary atherosclerosis	51	1.043	0.175	0.978	1.111
Graves’ disease							
	IVW	coronary atherosclerosis	12	1.039	0.002	1.013	1.066
	MR Egger	coronary atherosclerosis	12	1.068	0.034	1.013	1.127
	Weighted median	coronary atherosclerosis	12	1.043	0.016	1.009	1.078
Other types of hyperthyroidism							
	IVW	coronary atherosclerosis	10	1.055	0.021	1.008	1.104
	MR Egger	coronary atherosclerosis	10	1.133	0.055	1.016	1.263
	Weighted median	coronary atherosclerosis	10	1.088	0.001	1.035	1.144
TSH							
	IVW	coronary atherosclerosis	90	1.015	0.647	0.952	1.082
	MR Egger	coronary atherosclerosis	90	0.956	0.445	0.853	1.072
	Weighted median	coronary atherosclerosis	90	0.960	0.353	0.880	1.046
FT4							
	IVW	coronary atherosclerosis	16	0.990	0.815	0.910	1.077
	MR Egger	coronary atherosclerosis	16	1.024	0.792	0.860	1.218
	Weighted median	coronary atherosclerosis	16	1.023	0.749	0.887	1.180

A *p*-value less than 0.05 indicated statistical significance. SNPs, single nucleotide polymorphisms; OR, odds ratios; CI, confidence intervals; IVW, inverse variance weighting; FT4, free thyroxine; TSH, thyroid-stimulating hormone; MR, Mendelian Randomization.

**Table 2.** **Presents the findings on the variability and sensitivity of coronary atherosclerosis and thyroid function following the exclusion of invalid IVs**.

Exposure-Outcome	nSNP	MR Egger intercept		Cochran’s heterogeneity				Exposure-Outcome	nSNP	MR Egger intercept		Cochran’s heterogeneity			
		Intercept value	*p*	IVW-Q vaule	*p* (IVW)	Egger-Q value	*p* (Egger)			Intercept value	*p*	IVW-Q vaule	*p* (IVW)	Egger-Q value	*p* (Egger)
Hypothyroidism-coronary atherosclerosis	51	–0.013	0.068	106.941	5.1075 × 10^−⁢6^	99.858	2.45803 × 10^−⁢5^	Hypothyroidism-coronary atherosclerosis	51	–0.013	0.068	106.941	5.1075 × 10^−⁢6^	99.858	2.45803 × 10^−⁢5^
GD-coronary atherosclerosis	12	–0.011	0.267	10.208	0.512	8.826	0.549	GD-coronary atherosclerosis	12	–0.011	0.267	10.208	0.512	8.826	0.549
Other types of hyperthyroidism-coronary atherosclerosis	10	–0.023	0.200	14.738	0.098	11.847	0.158	other types of hyperthyroidism-coronary atherosclerosis	10	–0.023	0.200	14.738	0.098	11.847	0.158
TSH-coronary atherosclerosis	90	0.003	0.223	217.603	0.002	215.575	0.002	TSH-coronary atherosclerosis	90	0.003	0.223	217.603	0.002	215.575	0.002
FT4-coronary atherosclerosis	16	–0.002	0.666	59.309	0.428	59.115	0.398	FT4-coronary atherosclerosis	16	–0.002	0.666	59.309	0.428	59.115	0.398
Coronary atherosclerosis-hypothyroidism	31	0.017	0.372	230.156	9.80731 × 10^−⁢33^	223.800	5.66911 × 10^−⁢32^	coronary atherosclerosis-hypothyroidism	31	0.017	0.372	230.156	9.80731 × 10^−⁢33^	223.800	5.66911 × 10^−⁢32^
Coronary atherosclerosis-GD	31	0.008	0.735	45.826	0.032	45.643	0.025	coronary atherosclerosis-GD	31	0.008	0.735	45.826	0.032	45.643	0.025
Coronary atherosclerosis-other types of hyperthyroidism	31	0.013	0.478	34.326	0.268	33.724	0.249	coronary atherosclerosis-other types of hyperthyroidism	31	0.013	0.478	34.326	0.268	33.724	0.249
Coronary atherosclerosis-TSH	24	0.003	0.654	209.141	5.74538 × 10^−⁢32^	207.193	4.4248 × 10^−⁢32^	coronary atherosclerosis-TSH	24	0.003	0.654	209.141	5.74538 × 10^−⁢32^	207.193	4.4248 × 10^−⁢32^
Coronary atherosclerosis-FT4	24	0.007	0.075	40.284	0.014	34.748	0.041	coronary atherosclerosis-FT4	24	0.007	0.075	40.284	0.014	34.748	0.041

MR, Mendelian randomization; nSNP, the count of single nucleotide polymorphisms; IVW, inverse variance weighted; FT4, free thyroxine; TSH, thyroid-stimulating hormone; GD, Graves’ disease; IV, instrumental variables.

### 3.3 MR and Sensitivity Analysis for Causality Coronary Atherosclerosis on Thyroid Function

**Fig. 4.**
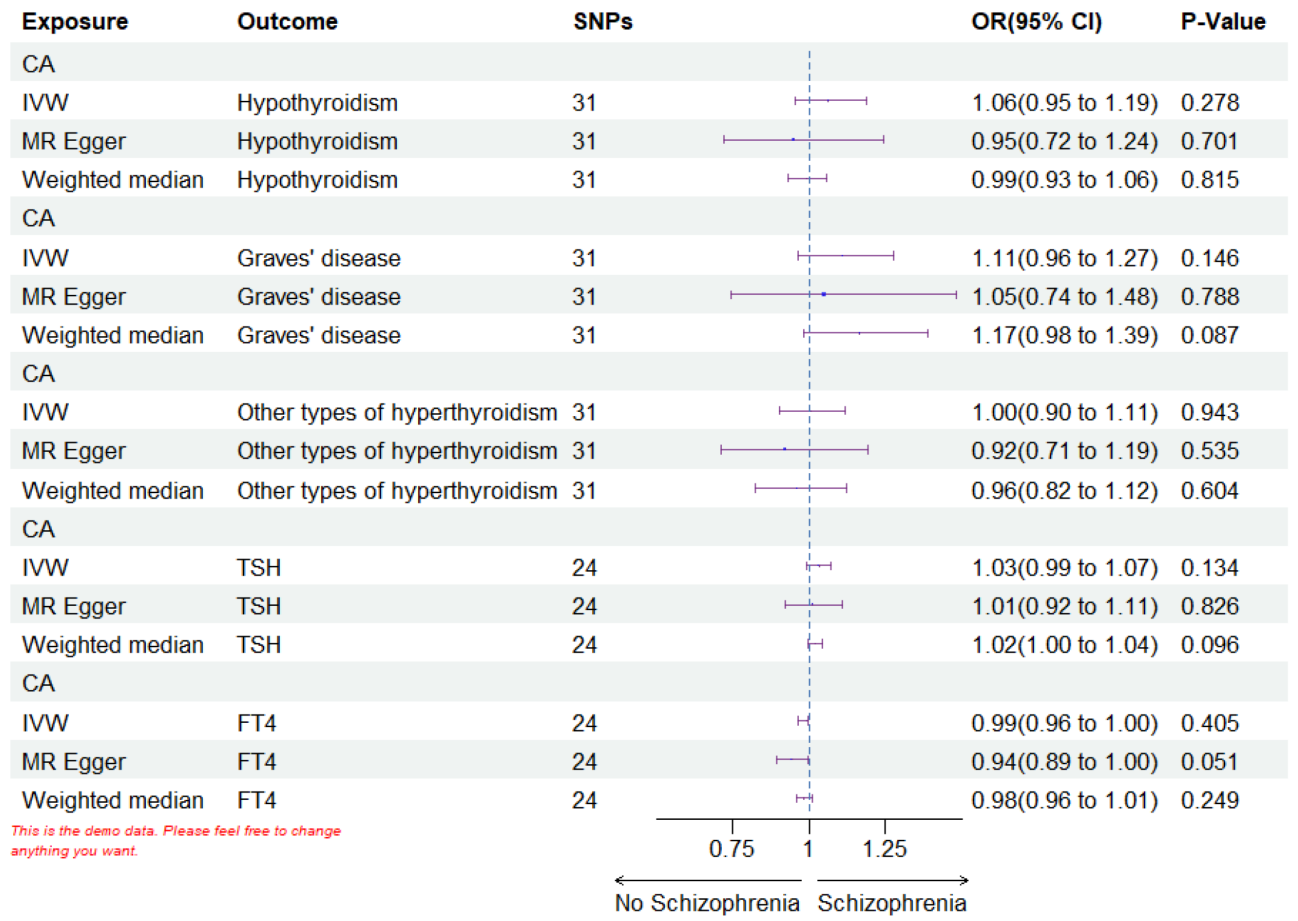
**The forest plots of reverse MR analysis**. IVW stands for inverse variance weighted. SNPs refers to the count of single nucleotide polymorphisms; OR, odds ratio; CI, confidence interval; CA, coronary atherosclerosis; FT4, free thyroxine; TSH, thyroid-stimulating hormone. Every statistical test was conducted using a two-tailed approach. A *p*-value less than 0.05 was deemed statistically significant. MR, Mendelian Randomization; SNPs, single nucleotide polymorphisms.

In the inverse Mendelian randomization study, coronary atherosclerosis was treated as the exposure, while five different thyroid function types were examined as the outcomes. After excluding all palindromic, heterozygous, duplicated, and weak SNPs, the counts included were 31 for hypothyroidism, 31 for GD, 31 for other hyperthyroidism types, 24 for TSH, and 24 for FT4. The inverse MR analysis found no causal link between coronary atherosclerosis and five thyroid function types using IVW, MR Egger, and Weighted Median methods (*p* > 0.05) (see Fig. 4 and Table 3). The scatter plots are shown in **Supplementary Fig. 1**. MR Egger intercepts did not measure horizontal pleiotropy for all reverse results (*p* > 0.05). The variability in SNPs related to hypothyroidism, GD, and TSH was investigated (*p* < 0.05) (see Table 2). Consequently, for the reverse MR analysis, we applied the IVW random-effects model to hypothyroidism, GD, and TSH, while the IVW fixed-effects model was employed for other hyperthyroidism types and FT4.

**Table 3.** **Presents the MR findings regarding the impact of coronary atherosclerosis on thyroid function**.

Exposure		Outcome	SNPs	OR	*p*-Value	95% CI Low	95% CI High
Coronary atherosclerosis							
	IVW	Hypothyroidism	31	1.063	0.278	0.952	1.187
	MR Egger	Hypothyroidism	31	0.948	0.701	0.722	1.243
	Weighted median	Hypothyroidism	31	0.992	0.815	0.931	1.058
Coronary atherosclerosis							
	IVW	Graves’ disease	31	1.108	0.146	0.965	1.274
	MR Egger	Graves’ disease	31	1.049	0.788	0.742	1.483
	Weighted median	Graves’ disease	31	1.166	0.087	0.981	1.386
Coronary atherosclerosis							
	IVW	Other types of hyperthyroidism	31	1.004	0.943	0.904	1.115
	MR Egger	Other types of hyperthyroidism	31	0.919	0.535	0.709	1.193
	Weighted median	Other types of hyperthyroidism	31	0.959	0.604	0.821	1.121
Coronary atherosclerosis							
	IVW	TSH	24	1.031	0.134	0.991	1.071
	MR Egger	TSH	24	1.011	0.826	0.921	1.108
	Weighted median	TSH	24	1.019	0.096	0.997	1.042
Coronary atherosclerosis							
	IVW	FT4	24	0.989	0.405	0.965	0.997
	MR Egger	FT4	24	0.942	0.051	0.891	0.997
	Weighted median	FT4	24	0.983	0.249	0.957	1.009

A *p*-value less than 0.05 indicated significance. SNPs, single nucleotide polymorphisms; OR, odds ratio; CI, confidence interval; IVW, inverse variance-weighted; TSH, thyroid-stimulating hormone; FT4, free thyroxine; MR, Mendelian Randomization.

Our conclusions are further validated by the leave-one-out analysis and the visualization outcomes. **Supplementary Figs. 2–7** visually presents the study’s findings through forest plots, leave-one-out sensitivity analyses, and funnel diagrams.

## 4. Discussion

The current bidirectional MR study analysis has identified a possible causal link between hypothyroidism, GD, hyperthyroidism, and a heightened risk of coronary atherosclerosis. These findings suggest that these conditions may act as genetic susceptibility factors in this process. Nevertheless, conditions like GD, and other forms of hyperthyroidism do not elevate the risk of coronary atherosclerosis. Moreover, no clear proof exists of a cause-and-effect link between FT4, TSH, and coronary atherosclerosis, whether in a direct or inverse manner.

Numerous research efforts have explored the effects of different clinical traits and examined the role of particular risk factors in CAD using MR. According to Wang *et al*. [28] who analyzed the causality of thyroid function on blood lipid index, they suggested that normal TSH levels were positively correlated to total cholesterol and negatively associated with low-density lipoprotein cholesterol, implying that abnormal thyroid function resulted in dyslipidemia. In addition, Ciofani *et al*. [29] conducted a comprehensive analysis that showed that low-density lipoprotein cholesterol and triglycerides increased the risk of ischemic heart disease in East Asian populations and Europeans by MR. It reinforced the concept that dyslipidemia was an important risk factor for CAD. Previous MR investigations proposed a potential association between thyroid function and CAD, warranting further exploration into this causal relationship. There were only two MR research studies that partially addressed the causality between thyroid function and coronary atherosclerosis or CAD. Larsson *et al*. [30] indicated a positive association between hypothyroidism and the likelihood of CAD in the UK Biobank study van Vliet *et al*. [31] demonstrated that genetic variation in TSH enhanced the risk of CAD. However, there were no MR researches analyzing the causality between thyroid function with coronary atherosclerosis. Low-grade coronary atherosclerosis is more insidious before it develops into CAD, so it is difficult to be clinically diagnosed and studied. At present, a more extensive collection of cohort data is required to comprehensively analyze the impact of hyperthyroidism, hypothyroidism, thyroxine, and thyrotropin on the development of coronary atherosclerosis. Therefore, we analyzed the causal influence of hyperthyroidism and hypothyroidism on coronary atherosclerosis patients in FinnGen cohorts. A recent GWAS meta-analysis [21] featured in Nature Communications involved 271,040 euthyroid participants of European descent, as part of ThyroidOmics Consortium study. Subsequently, we conducted this MR analysis using TSH and FT4 GWAS data from the recently-summarized loci associated with thyroid function.

Our research indicated that individuals genetically predisposed to hypothyroidism have an increased likelihood of developing coronary atherosclerosis, and similar observational studies corroborated our conclusions. Among the 412 patients with hypothyroidism conducted in Saudi Arabia, a total of 21.8% were diagnosed with CAD, indicating a higher prevalence of CAD among individuals with hypothyroidism compared to the general population [32]. Auer *et al*. [33] suggested that patients diagnosed with hypothyroidism exhibited a notably increased risk of CAD compared to individuals with normal thyroid function or hyperthyroidism. However, observational studies were utilized to initially identify risk factors and it was difficult to distinguish unavoidable confounding factors and undefined two-way causality [34]. Hypothyroidism seemed to mediate some potential confounding factors and regulate coronary atherosclerosis, including serum retinol-binding protein 4, fatty liver, and coronary endothelial dysfunction [35, 36, 37]. However, the random distribution of genetic variation and genotype at birth have determined the priority of the MR analysis [38, 39]. Although GD shared similar clinical and diagnostic features with other types of hyperthyroidism, a genome-wide linkage and association study have identified significant and unique effects on GD susceptibility from gene variants such as *HLA*, *CTLA4*, and *PTPN22* [40]. Therefore, this MR research explored the causal relationship between the two phenotypes and coronary atherosclerosis. Our research revealed that both GD and other forms of hyperthyroidism elevated the likelihood of developing coronary atherosclerosis. A study observing 744 patients who had coronary angiography found that those with both overt and subclinical hyperthyroidism showed more severe stenoses (39.2% vs 37.8% vs 24.2%; *p* = 0.007) and higher coronary calcium scores (456.5 vs 199.5 vs 155.9; *p* < 0.0001) compared to individuals with normal thyroid function [41]. Numerous case studies have shown a higher occurrence of CAD in individuals with GD, leading to coronary spasm [42, 43, 44, 45]. Our MR analysis was largely consistent with the results of current observational studies and provided unidirectional causality and avoided confounding factors.

Besides examining hyperthyroidism and hypothyroidism, we performed MR analyses on people with typical TSH and FT4 levels to determine if genetic variations in these hormones are linked to a higher risk of coronary atherosclerosis. The reference ranges are typically determined by calculating the upper and lower 2.5th percentiles of the TSH distribution [19]. No significant causal relationship between coronary atherosclerosis and TSH or FT4 was observed in this MR study. While hyperthyroidism and hypothyroidism are linked to a higher chance of coronary atherosclerosis, the findings from TSH and FT4 tests indicate that normal TSH and FT4 levels do not entirely eliminate this risk. Our findings are aligned with previous observational studies and MR analysis. Based on the analysis of the most extensive CAD dataset available, Marouli *et al*. [46] reported no substantial proof linking TSH or FT4 levels to the likelihood of developing CAD. Our research suggests that FT4 and TSH levels are not directly associated with the genetic, immune, and environmental factors that lead to coronary atherosclerosis. Instead, these levels appear to predominantly reflect the functional status of the thyroid and pituitary glands. This finding indicated that these two hormones may not be major contributors to the genetic susceptibility associated with coronary atherosclerosis.

This study offered indicative findings for treating patients with hypothyroidism, GD, and other types of hyperthyroidism, revealing an increased genetic predisposition to coronary atherosclerosis. The results suggested that individuals with irregular thyroid activity need to be cautious about the onset and progression of coronary atherosclerosis and should have comprehensive medical check-ups. It is imperative for these patients to pay attention to and control their status of SMuRFs to mitigate the risk of CAD. Furthermore, we anticipate that these results will stimulate other CAD research groups to conduct relevant randomized controlled trials to complement the implementation of SMuRFless standards and mitigate their incidence of CAD.

This MR research has a number of constraints. To begin with, findings from European groups do not accurately reflect the worldwide scenario regarding causal links. Future comprehensive studies involving different ethnic groups are needed. Meanwhile, the presence of heterogeneity among certain exposed SNPs introduces some variability, thereby attenuating the significance of the MR results. Additionally, there are many subtypes of thyroid hormones, and we did not analyze all of them due to the available SNP data in the database, which will require further study.

## 5. Conclusions

To conclude, this two-way MR study explored a genetic tendency towards hypothyroidism, GD, and various hyperthyroid conditions, which were shown to elevate the likelihood of coronary atherosclerosis. Nonetheless, typical levels of TSH and FT4 showed no association with the likelihood of coronary atherosclerosis. Therefore, for patients with hyperthyroidism or hypothyroidism, it is crucial to prioritize routine screening on coronary atherosclerosis to avoid the occurrence of CAD. These findings provide insights into the pathogenesis and risk factors associated with coronary atherosclerosis and reinforce the establishment of SMuRFless in CAD.

## Availability of Data and Materials

The data can be obtained from the corresponding author via reasonable request.

## Supplementary Material

Supplementary material associated with this article can be found, in the online version, at https://doi.org/10.31083/j.rcm2512453.
