# Supplementary material for: A Two-Way Mendelian Randomization Analysis on the Link between Thyroid Activity Function and Coronary Atherosclerosis
Source: Rev Cardiovasc Med. 2024 Dec 24;25(12):453. doi: 10.31083/j.rcm2512453 (PMC11683704; doi:10.31083/j.rcm2512453)
Supplement: Supplementary file 1 [file 2153-8174-25-12-453-s1.zip › attachment2-Supplement figures.docx]

**Supplementary Materials**

**SUPPLEMENTARY NOTES**

**Supplementary Figure 1 |** The forest plots for causal effect of thyroid function on coronary atherosclerosis.

**Supplementary Figure 2 |** The Leave-one-out sensitivity analysis for causal effect of thyroid function on coronary atherosclerosis.

**Supplementary Figure 3 |** The funnel plot of individual SNP effects of thyroid function on coronary atherosclerosis.

**Supplementary Figure 4 |** The forest plots for causal effect of coronary atherosclerosis on thyroid function.

**Supplementary Figure 5 |** The Leave-one-out sensitivity analysis for causal effect of coronary atherosclerosis on thyroid function.

**Supplementary Figure 6 |** The funnel plot of individual SNP effects of coronary atherosclerosis on thyroid function.

**Supplementary Figure 7 |** The scatter plots for the causal effect of reverse MR analysis.

**Supplementary Figure 1 |** The forest plots for causal effect of thyroid function on coronary atherosclerosis(CA).


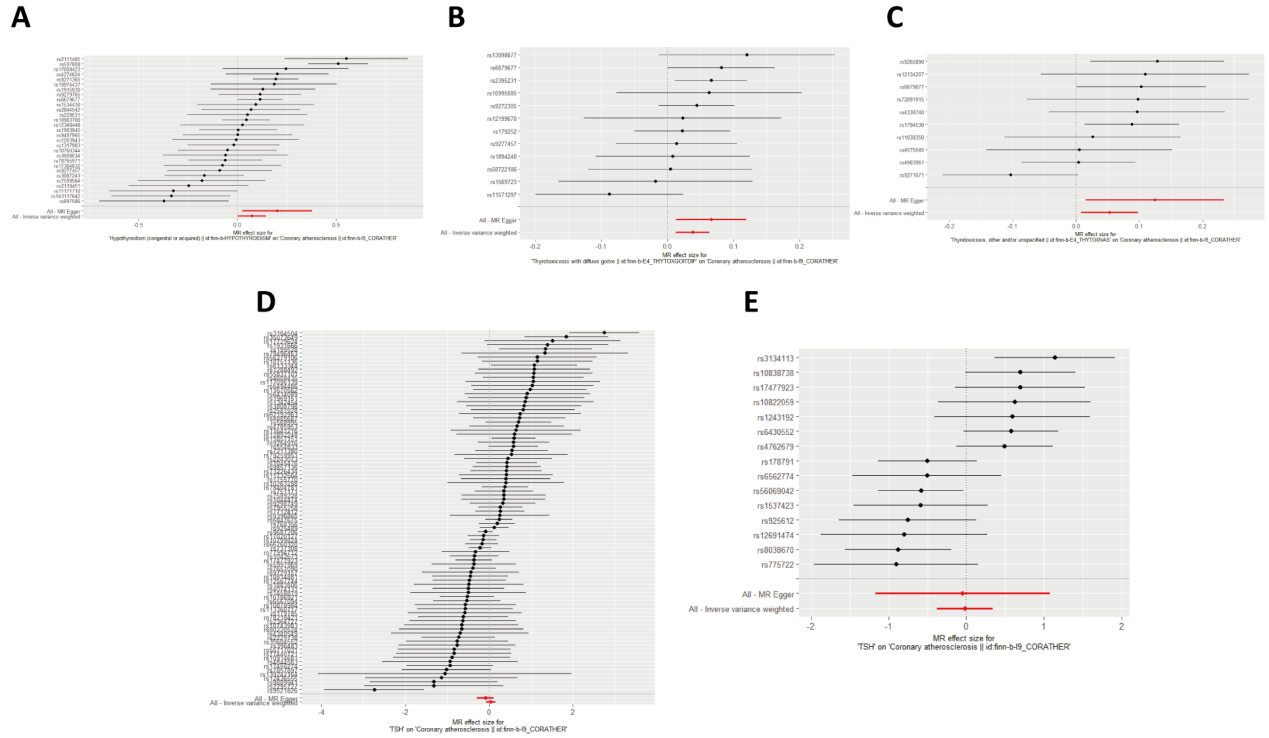


1. hypothyroidism on CA, (b) hyperthyroidism with diffuse goitre on CA, (c) other unspecified hyperthyroidism on CA, (d) TSH on CA, (e) FT4 on CA.

**Supplementary Figure 2 |** The Leave-one-out sensitivity analysis for causal effect of thyroid function on coronary atherosclerosis(CA).

1. hypothyroidism on CA, (b) hyperthyroidism with diffuse goitre on CA, (c) other unspecified hyperthyroidism on CA, (d) TSH on CA, (e) FT4 on CA.


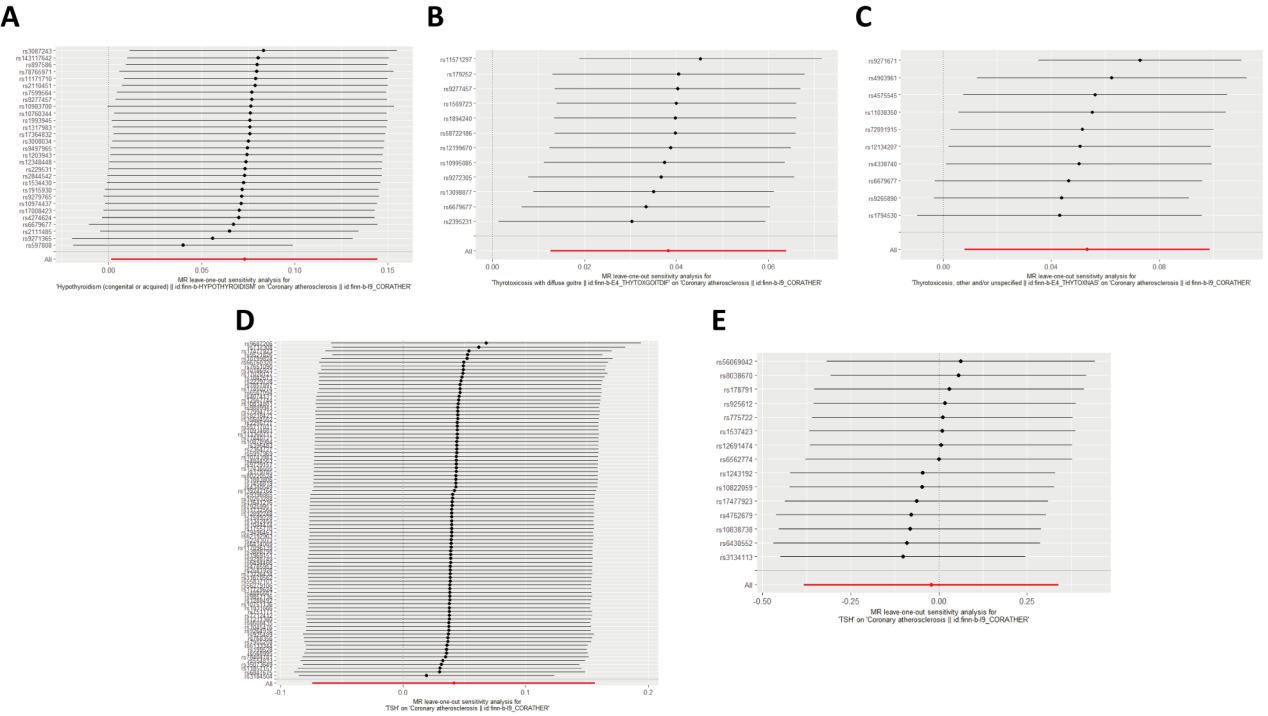


**Supplementary Figure 3 |** The funnel plot of individual SNP effects of thyroid function on coronary atherosclerosis(CA).


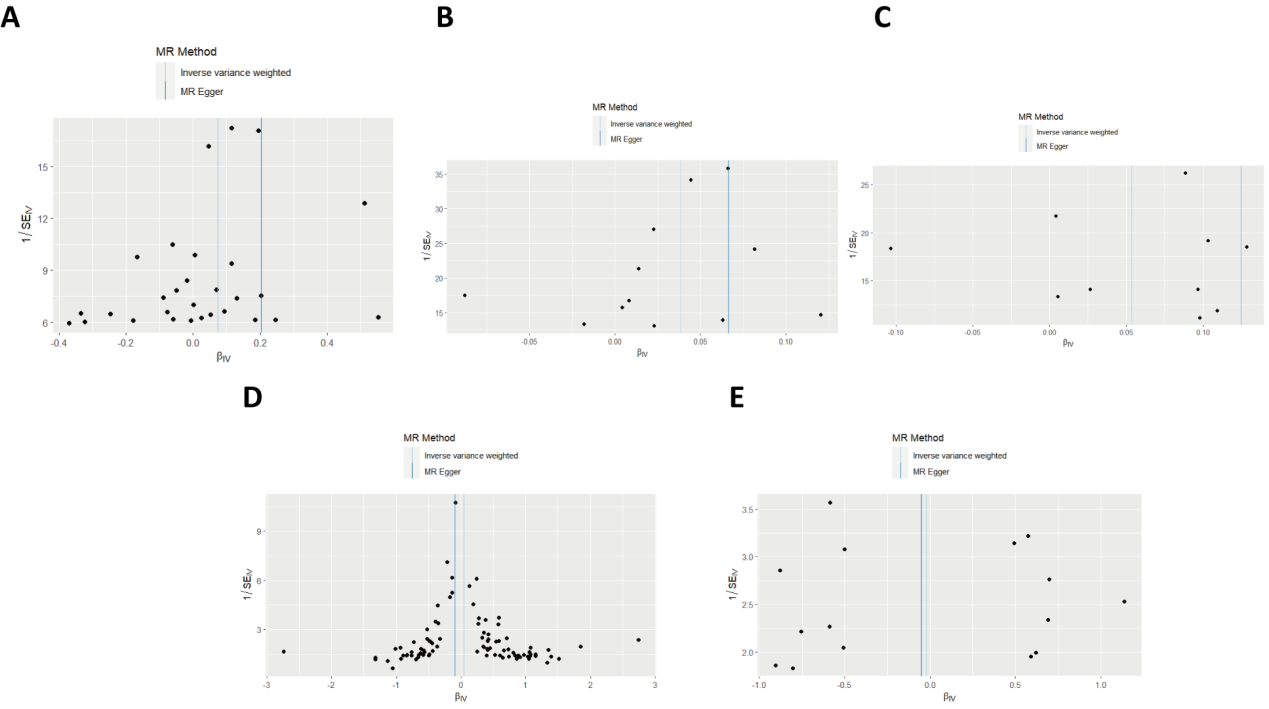


1. hypothyroidism on CA, (b) hyperthyroidism with diffuse goitre on CA, (c) other unspecified hyperthyroidism on CA, (d) TSH on CA, (e) FT4 on CA.

**Supplementary Figure 4 |** The forest plots for causal effect of coronary atherosclerosis(CA) on thyroid function.


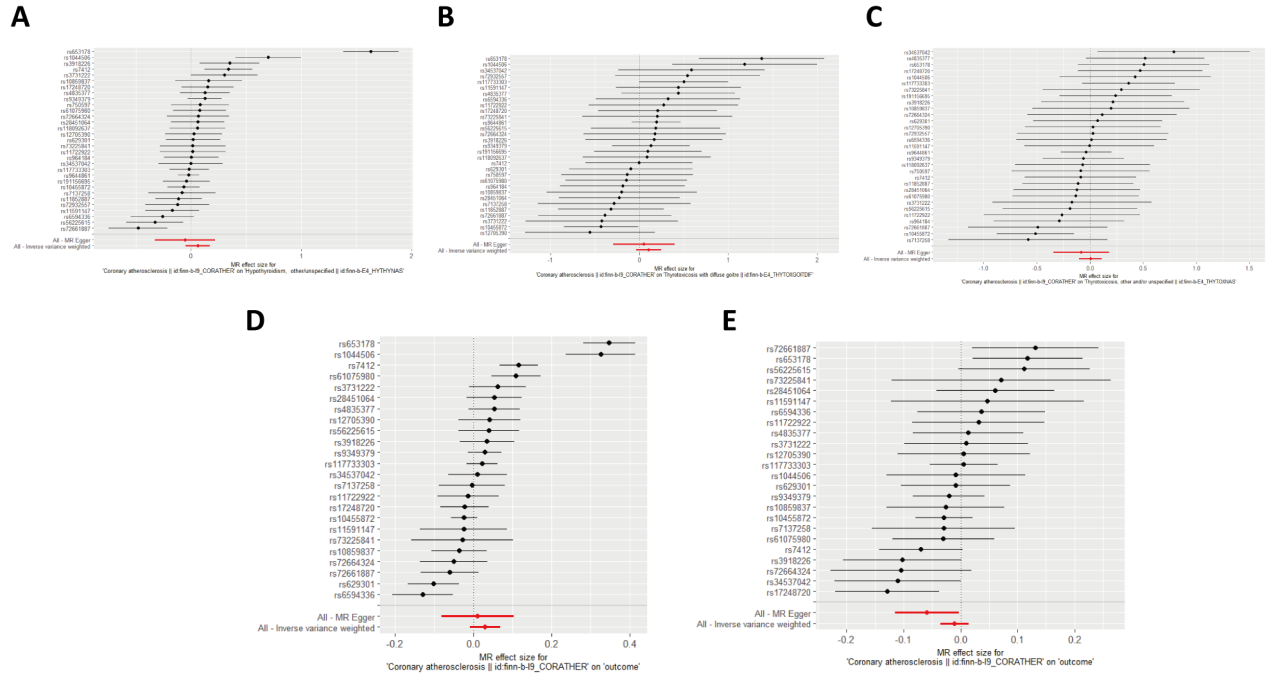


(A) CA on hypothyroidism. (B) CA on Graves' disease. (C) CA on other types of hyperthyroidism. (D) CA on thyroid-stimulating hormone. (E) CA on free thyroxine.

**Supplementary Figure 5 |** The Leave-one-out sensitivity analysis for causal effect of coronary atherosclerosis(CA) on thyroid function.


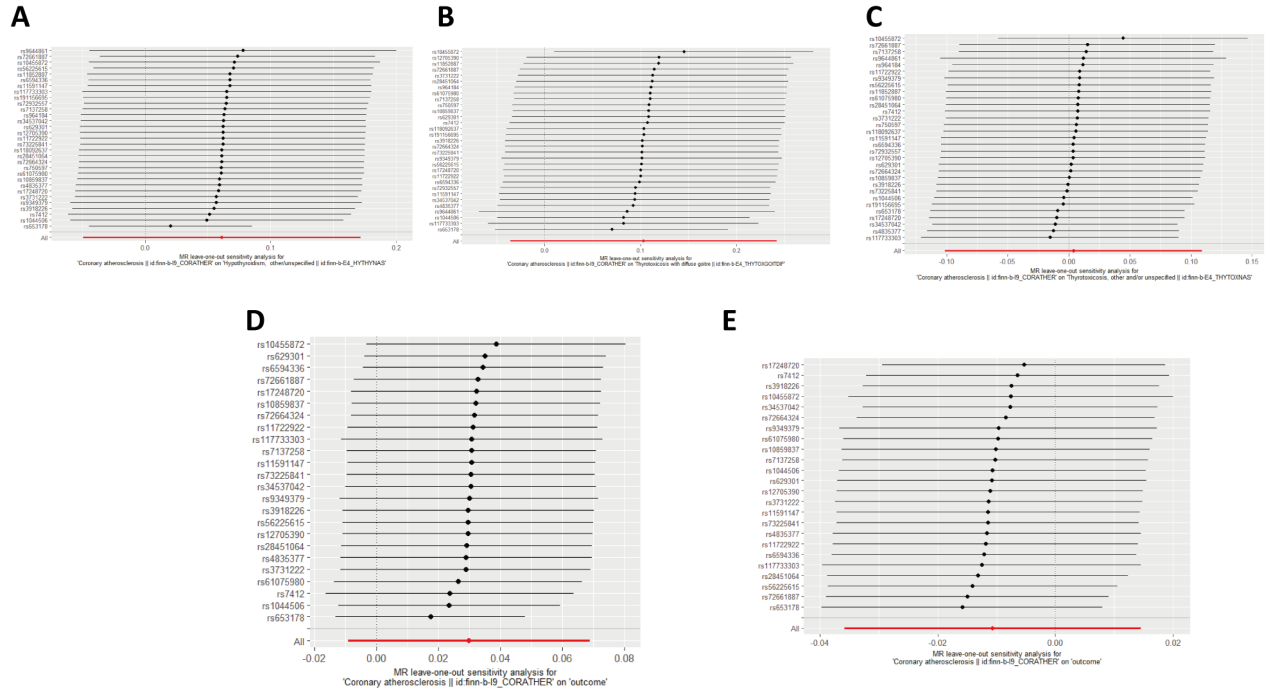


(A) CA on hypothyroidism. (B) CA on Graves' disease. (C) CA on other types of hyperthyroidism. (D) CA on thyroid-stimulating hormone. (E) CA on free thyroxine.

**Supplementary Figure 6 |** The funnel plot of individual SNP effects of coronary atherosclerosis(CA) on thyroid function.


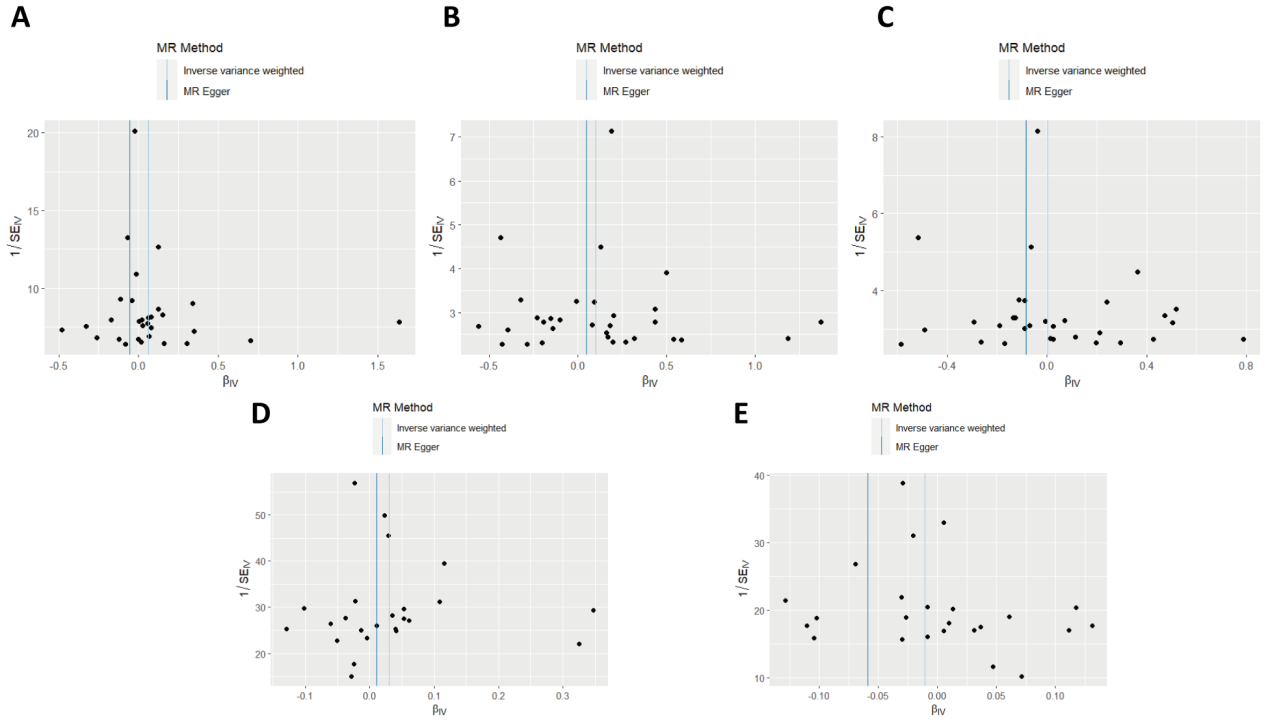


(A) CA on hypothyroidism. (B) CA on Graves' disease. (C) CA on other types of hyperthyroidism. (D) CA on thyroid-stimulating hormone. (E) CA on free thyroxine.

**Supplementary Figure 7 |** The scatter plots for the causal effect of reverse MR analysis.


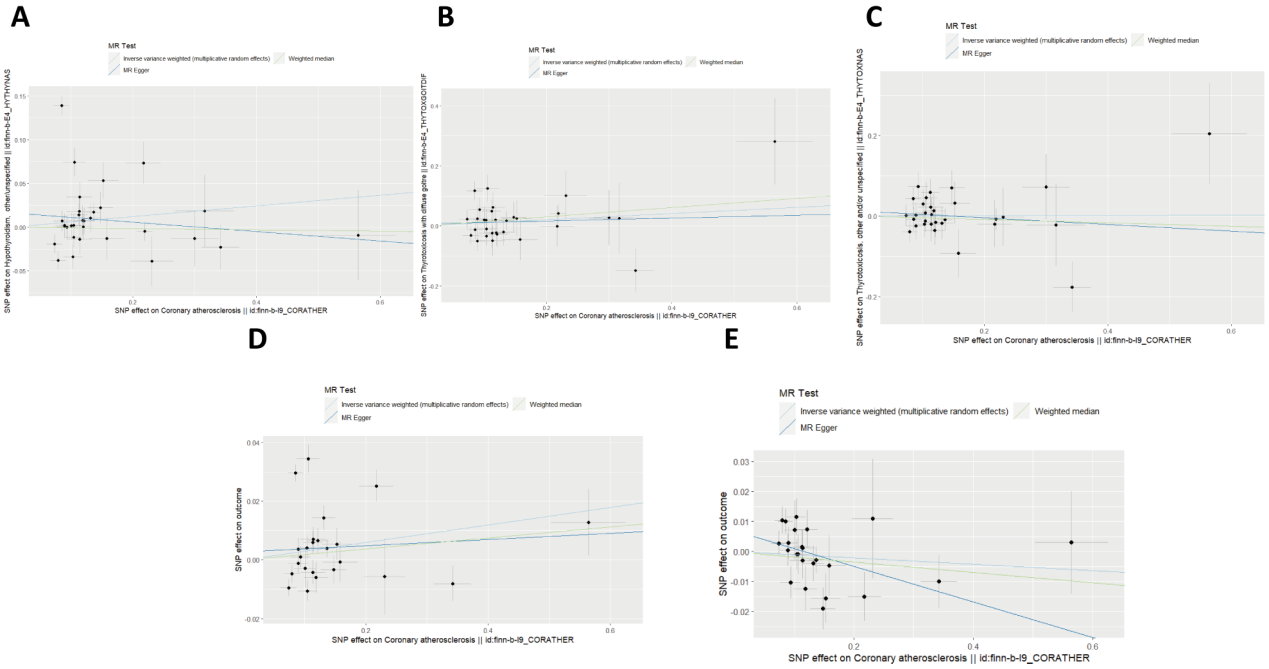


(A) CA on hypothyroidism. (B) CA on Graves' disease. (C) CA on other types of hyperthyroidism. (D) CA on thyroid-stimulating hormone. (E) CA on free thyroxine.
